# Bimetallic Metal-Organic Framework Derived Metal-Carbon Hybrid for Efficient Reversible Oxygen Electrocatalysis

**DOI:** 10.3389/fchem.2019.00747

**Published:** 2019-11-08

**Authors:** Yu Zhou, Yan Zhang, Xianzhen Xu, Shenlong Zhao, Ziyi Guo, Kuang-Hsu Wu, Chunhui Tan, Zonghua Wang

**Affiliations:** ^1^Shandong Sino-Japanese Center for Collaborative Research of Carbon Nanomaterials, College of Chemistry and Chemical Engineering, Qingdao University, Qingdao, China; ^2^School of Chemical and Biomolecular Engineering, The University of Sydney, Sydney, NSW, Australia; ^3^School of Chemical Engineering, The University of New South Wales, Kenginton, NSW, Australia

**Keywords:** metal-organic framework, NiCo alloy composites, bi-functional electrocatalyst, oxygen reduction reaction, oxygen evolution reaction

## Abstract

Development of cost-effective electrocatalysts for oxygen reduction reaction (ORR) and oxygen evolution reaction (OER) is key to enabling advanced electrochemical energy conversion technologies. Here, a novel nitrogen-doped metal-carbon hybrid (NiCo/CN) with a unique 3D hierarchical structure, consisting of uniformly distributed bimetallic nanoparticles encapsulated by partially graphitized N-doped carbon shells, is fabricated by a one-step pyrolysis of a nanoscale metal-organic framework as precursor, which exhibits excellent activity for both ORR and OER. The surface chemical changes on the carbon hybrid probed by X-ray photoelectron spectroscopy (XPS) reveal the presence of favorable electronic interaction at the metal-nitrogen-carbon interface. Remarkably, the NiCo/CN catalyst prepared at high temperature (800°C) manifests a comparable performance to a commercial Pt/C catalyst for the ORR, but also superior stability, path selectivity and methanol tolerance. On the other hand, the *E*_onset_ (1.48 V vs. reversible hydrogen electrode) and ${E_{\text{j}=10\text{mA/cm}}}^{2}$ of NiCo/CN-800 for OER is very close to the state-of-the-art noble catalyst RuO_2_ (E_onset_ = 1.46 and ${E_{\text{j}=10\text{mA/cm}}}^{2}$) along with superior stability over 20 h of operation. The excellent catalytic property is attributable to the unique nanostructure, high porosity and the constructive synergistic effects of the elements M, N, and C.

## Introduction

Reversible oxygen reactions, including the oxygen reduction reaction (ORR) and oxygen evolution reaction (OER), are becoming increasingly important for many renewable conversion technologies and other important industrial processes (Pei et al., 2017; Gong et al., 2018; Zheng et al., 2019). Current benchmark catalysts for the ORR and OER are, respectively, Pt-based and Ir/Ru-based materials. However, the high cost and scarcity of the noble metal substances greatly limit their wide applications (Hao et al., 2015). Furthermore, noble metal-based catalysts often suffer from multiple other drawbacks, including poor durability, impurity poisoning, and low selectivity, with detrimental effects to the environment on unregulated disposal (Zhao et al., 2019). Therefore, it is highly desirable to develop alternative ORR and OER catalysts with low cost (i.e., non-noble materials) and high performance for the sustainable energy technologies (Feng et al., 2016a,b).

Among various non-noble materials, heteroatom-doped nanocarbon hybrid materials with metal oxides and hydroxides manifested huge potential to be high-performance bifunctional electrocatalysts (Morozan et al., 2011). In particular, the doped nanocarbons with metal–nitrogen–carbon (M–N–C) centers attracted wide interest due to their excellent catalytic activity and durability. Recent reports indicated that nitrogen-doped M–N–C hybrid catalysts can be compensable for the intrinsic deficiency in conductivity to a considerable extent, providing more efficient electron transfer between the catalytic sites and intermediates (Xia et al., 2015; Liu and Dai, 2016; You et al., 2016). Density functional theory (DFT) calculations further revealed that upon oxygen binding, there is a partial electronic interaction between the empty *d*_z_ orbitals of metal and the antibonding orbitals of oxygen, facilitating the subsequent catalytic steps (Artyushkova et al., 2017). Unfortunately, due to the low exposure of active sites and efficient mass transport, their overall performance is yet to meet the demand for a practical oxygen electrode.

Metal-organic frameworks (MOFs), as a new family of porous crystalline materials formed via self-assembly of metal ions and organic ligands (Furukawa et al., 2013; Zhu and Xu, 2014; Gao et al., 2018; Li C. et al., 2019; Li Y. et al., 2019), provide an ideal structural platform to create advanced metal-carbon hybrid electrocatalysts with unique properties, such as chemical composition versatility, high porosity and surface area (Ma et al., 2014a,b, 2016). However, due to the poor thermal stability of the single metal nodes, MOF-derived carbons usually suffer from a significantly reduced specific surface area that limits their catalytic application (Shui et al., 2015). Recently, it was shown that partial replacement of metal ions in the MOF framework by a second metal species with similar nature can not only retain their original structure but also endow more functionalities (Wang et al., 2016; Zhao et al., 2016; Fu et al., 2017). Therefore, designing bi-/multi-metallic MOF derivatives with favorable chemical compositions and desirable structure can be a promising strategy for developing cost-effective noble-metal-free electrocatalysts that can deliver a catalytic performance comparable to, or even better than, noble metal catalysts.

As reported, Cobalt (Co)—especially the Co–Nx species—can greatly improve the oxygen reduction activity by reducing the adsorption of oxygen containing intermediates (Zhang et al., 2019). On the other hand, Ni is regarded as the most active transition metal for electrocatalytic oxygen evolution. Therefore, bimetallic carbon nanomaterial with Co and Ni could be an efficient electrocatalyst for both ORR and OER (Wang et al., 2018). Here, a porous heteroatom-doped carbon with the M-N-C structure derived from bimetallic MOFs was fabricated and used as bifunctional electrocatalysts for both ORR and OER. The structure and component of the as-prepared NiCo/CN-800 are characterized by scanning electron microscopy (SEM), transmission electron microscopy (TEM), X-ray diffraction (XRD), and nitrogen adsorption isotherm. X-ray photoelectron spectroscopy (XPS) was also conducted to investigate the surface chemical states of the NiCo/CN-800 and the electronic interaction between M, N, and C atoms. Remarkably, the as-prepared NiCo/CN-800 exhibited similar catalytic activity but superior stability with prominent methanol tolerance to a commercial 20 wt% Pt/C catalyst for the ORR. Moreover, the hybrid catalyst could deliver an OER with an onset potential of 1.48 V (vs. reversible hydrogen electrode, RHE) and an over-potential of only 350 mV to achieve a stable current density of 10 mA cm^−2^ for over 20 h testing. The outstanding ORR and OER catalytic properties are ascribed to the unique nanostructure, high porosity, and electronic synergistic effects of the elements M, N, and C. This concept provides a new strategy to design heteroatom-doped porous carbon-based nanomaterials for energy conversion applications including catalysis, supercapacitors, and rechargeable batteries.

## Experimental

### Chemicals

Nafion solution (5%), platinum on graphitized carbon (Pt/C, 20 wt%), was obtained from Sigma-Aldrich. 2-Methylimidazole (C_4_H_6_N_2_), Cobalt nitrate hexahydrate (Co(NO_3_)_2_·6H_2_O), nickel nitrate (Ni(NO_3_)_2_·6H_2_O), Polyvinyl -pyrrolidone (PVP) and Triethylamine (C_6_H_15_N) were purchased from Aladdin. Methanol and hydrochloric acid were obtained from Shanghai Chemical Reagent Inc. All chemicals were used without further purification.

### Synthesis of NiCo/ZIF-67 Nanooctahedra

Zeolitic imidazolate framework- 67 (ZIF-67) was prepared by the following procedure: Co(NO_3_)_2_·6H_2_O (5.88 g) and PVP (5 g) were dissolved in 500 mL methanol to form solution A. 2-Methylimidazole (6.626 g) and triethylamine (0.4 mL) were dissolved in another 500 mL methanol to form solution B. Solution B was then poured into solution A for 10 min under magnetic stirring. Subsequently, the mixed solution was aged at room temperature for 24 h. The sample was collected by centrifugation and washed with methanol three times. Finally, the sample (ZIF-67) was dried under vacuum at 100°C for 24 h (Zhang et al., 2017).

A tunable *in-situ* doped bimetallic ZIF-67 (Ni-Co) was also prepared, and the specific steps are as follows: preparing solution A: Co(NO_3_)_2_·6H_2_O, Ni (NO_3_)_2_·6H_2_O (total 5.88 g). The doping ratio was 10, 20, 30, 40, 50, 60, 70, 80, and 100%) and PVP (5 g) was dissolved in 500 mL methanol to form solution A. The rest of the processes were identical to the above process.

### Synthesis of the Series of NiCo/CN-n Composites

The as-prepared NiCo/ZIF-67 was pyrolyzed to NiCo/CN-n composites. A total of 500 mg NiCo/ZIF-67 powers was placed in a silica boat and carbonized at 600, 700, 800, and 900°C in a tube furnace. The carbonized temperature was elevated with a heating speed of 5°C min^−1^ and maintained for 3 h under Argon atmosphere with a gas flow of 50 mL min^−1^. Then, all those products were immersed in hydrochloric acid for 12 h. After centrifugation, the NiCo/CN-600, NiCo/CN-700, NiCo/CN-800, and NiCo/CN-900 products were obtained at 600, 700, 800, and 900°C.

### Characterization

The morphologies of the power were obtained by SEM images on Nova NanoSEM 450. Transmission electron microscope (TEM) images were taken by Tecnai G2 F20. The Powder XRD patterns were obtained by using a D-MAX 2500/PC with a graphite-monochromated Cu Kα radiation source. XPS was obtained by using a ThermoFisher ESCALAB 250xi. Raman spectrum was measured on a LabRAM HR-800 Raman spectrometer. The nitrogen adsorption and desorption isotherms measurements were carried out by AutoChemi II 2920 at 77 K.

### Electrochemical Measurements

A conventional three-electrode was used for electrochemical measurements by using a VSP-300 electrochemical workstation (Bio-Logic SAS, France) at 25°C. A total of 0.3 mL alcohol, 0.3 mL deionized water, 0.05 mL Nafion solution (5 wt%), and 5 mg NiCo/CN-n or Pt/C catalysts was adequately mixed together by ultrasonic agitation. Next, 8 uL of the mixture was dropped onto the clear glassy carbon disk electrode (diameter = 0.4 cm), which was used as a working electrode. A platinum wire and an Ag/AgCl were utilized as a counter electrode and a reference electrode, respectively. During the measurement, O_2_-saturated alkaline M KOH aqueous solution was used as the electrolyte.

Cyclic voltammetry (CV) was scanned at a rate of 50 mV·s^−1^ in the potential range from 0 to −0.8 V. The rotating disk electrode (RDE) measurement used varying speeds from 400 to 2,025 rpm at a scanning rate of 5 mV·s^−1^. The rotating ring-disk electrode (RRDE) measurement was used at a rotating speed of 1,600 rpm at a scanning rate of 5 mV·s^−1^ in O_2_-saturated 0.1 M KOH aqueous solution. The electron transfer number (*n*) was calculated on the basis of the Koutecky-Levich equation (Naveen et al., 2017):

(1) $$\begin{array}{r} 1/J=1/J_{\text{L}}+1/J_{\text{K}}\text{=}1/B\omega^{0.5}+1/J_{\text{K}} \end{array}$$

(2) $$\begin{array}{r} B=0.62nFC_{0}D_{0}^{2/3}\nu^{-1/6} \end{array}$$

where *J* was the measured current density, *J*_K_ and *J*_L_ were the kinetic- and diffusion-limiting current densities, ω was the angular velocity, *F* was the Faraday constant, *C*_0_ was the bulk concentration of O_2_ and *v* was the kinematic viscosity of the electrolyte.

Another efficient method to estimate the electron transfer number (*n*) was based on the RRDE measurements, with the following equation (Naveen et al., 2017):

(3) $$\begin{array}{r} n=4I_{\text{d}}\left(I_{\text{d}}\text{+}I_{\text{r}}/N\right) \end{array}$$

where *I*_d_ is disk current and *I*_r_ the ring current, and *N* is the current collection efficiency (0.43) for the Pt ring.

## Results and Discussion

### Synthesis, Structure, and Composition of Composites

ZIF-67 was synthesized by the solvothermal reaction (Zhang et al., 2017), and the as-prepared sample was chosen as a precursor for ORR/OER electrocatalyst. Then, NiCo/ZIF-67 (replace metal nickel by *in-situ* replacement from ZIF-67) was transformed into the NiCo/CN via carbonization and acidification. The precursors were pyrolyzed at 600, 700, 800, and 900°C, namely NiCo/CN-600, NiCo/CN-700, NiCo/CN-800, and NiCo/CN-900, respectively. At the same time, the proportion of bi-metal was adjusted to further explore the electrocatalytic properties. As shown in the SI (Supporting Information) the LSV of the different doping ratios and different pyrolysis temperatures is compared in Figure S1 and NiCo/CN (40%) at different pyrolysis temperatures is shown in Figure S2. The results show that when the doping ratio is around 40% and the pyrolysis temperature is about 800°C, the obtained catalyst exhibits the highest electrocatalytic activities among all the samples. So, the doping ratio (40%) and the pyrolysis temperature (800°C) were chosen to synthesize in this work.

**Figure 1:**
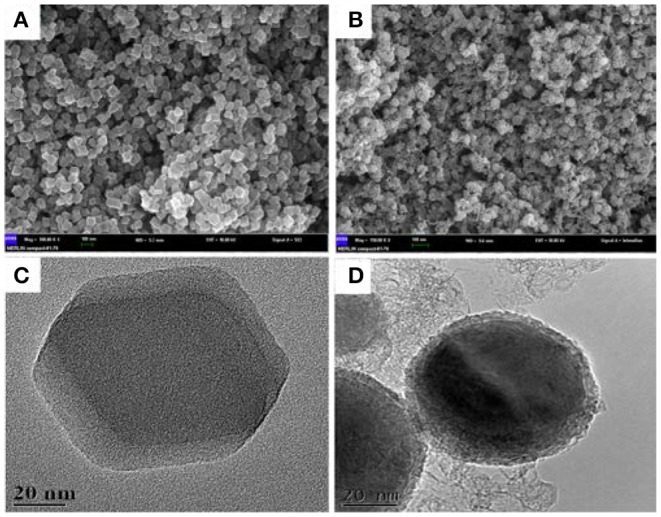
SEM **(A,B)** and TEM **(C,D)** image of NiCo/ZIF-67 **(A,C)**, and NiCo/CN-800 **(B,D)**.

Morphology of the as-prepared NiCo/ZIF-67 and its derivative (NiCo/CN-800) were characterized by SEM and TEM techniques (Figure 1). Figures 1A,C show that NiCo/ZIF-67 possesses a polygonal three-dimensional structure with an average size of ~90 nm. High-resolution TEM images in Figures 1B,D indicate that the three-dimensional structure was well preserved after pyrolysis. Compared with Figure S3, the as-prepared NiCo/ZIF-67 well inherits the morphology and structural feature of ZIF-67 (Wang et al., 2013; Zhang et al., 2017). This translated structure laid a good foundation for electrocatalysis at the heterogeneous surface. To investigate the component of the as-prepared catalysts, TEM mapping and energy dispersive X-ray (EDX) microanalysis are carried out. As show in Figure S4 the TEM-mapping images show C, N, O, Co, and Ni elements are well dispersed on the surface of NiCo/CN-800. Besides, TEM-EDX microanalysis indicates the content is 88.16, 5.66, 3.78, 2.04, and 0.36% for C, N, O, Co, and Ni, respectively, which is consistent with the result of XPS.

**Figure 2:**
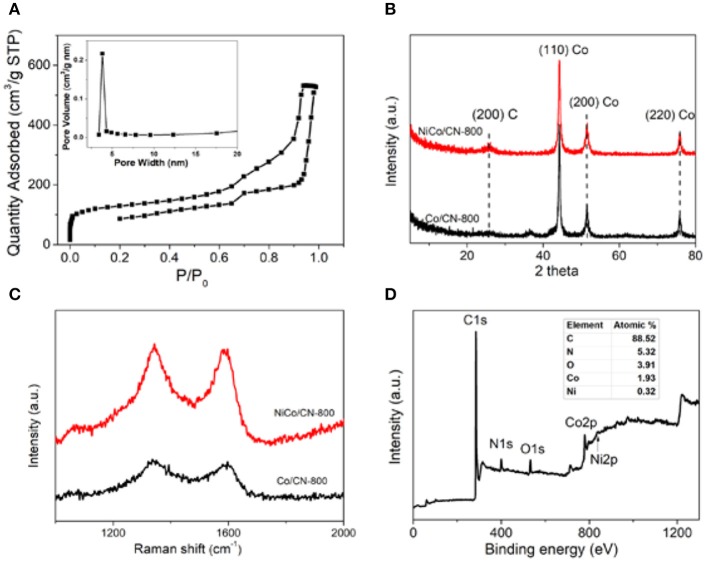
**(A)** N_2_ adsorption-desorption isotherm of NiCo/CN-800; inset is the corresponding pore size distribution. **(B)** XRD patterns of the Co/CN-800 and NiCo/CN-800. **(C)** Raman patterns of the Co/CN-800 and NiCo/CN-800. **(D)** Survey spectrum of NiCo/CN-800.

The specific surface area and pore structure of NiCo/CN-800 were investigated by N_2_ adsorption isotherms. In Figure 2, the isotherm of the as-prepared NiCo/CN-800 sample shows a typical profile for a mesoporous material, and the specific surface area was found to be 558 m^2^/g. The pore distribution indicates that the pore size is about 3.8 nm (inset of Figure 2). The isotherm of the as-prepared NiCo/ZIF-67 sample is shown in Figure S5. The specific surface area was found to be 408 m^2^/g. Clearly, the MOF derivative has a large specific surface area and a desirable pore size distribution, retained after pyrolysis. These advantages are derived from the inherent characteristics of the NiCo/ZIF-67 precursor, which would benefit the mass transport during the electrocatalysis. In addition, the N_2_ adsorption-desorption isotherm of the carbonized product of Ni-ZIF and ZIF-67 are shown in Figure S6. The presence of crystalline species was characterized by XRD. Figure 2B shows that there are some distinct characteristic peaks of inorganic carbon and metal crystal lattice. NiCo/CN-800 has a large amount of disordered graphitic carbon (200) at 2θ = 25° after high temperature treatment, with some metallic species as indicated by the (110), (200), and (220) crystal planes (Hou et al., 2015, 2016). The degree of graphitization was measured by Raman spectroscopy as shown in Figure 2C. The peak at 1,590 cm^−1^ is ascribed to the sp^2^ vibration in the graphite layer, or the G band. The peak at 1,320–1,330 cm^−1^ is assigned to the sp^3^ vibration of the amorphous carbon structure, or the D band. The ratio of the relative intensities of the two peaks, *I*_D_/*I*_G_, reflects the level of graphitization of both degree of disorder and degree of defects. The I_D_/I_G_ ratio was calculated to be 0.89 and 0.98 for Co/CN-800 and NiCo/CN-800, respectively. That is, after doping with Ni, the disorder degree of carbon is improved, and the NiCo/CN-800 composites have more defect structures, which can provide electrocatalysis active sites, and is beneficial to the conduction of electrons (Liu et al., 2016).

To further explore the composition and structure of NiCo/CN-800, XPS was conducted to analyze the element types and scales. As shown in Figure 2D, NiCo/CN-800 is mainly composed of five elements: C, N, O, Co, and Ni accounting for 88.52, 5.32, 3.91, 1.93, and 0.32% (inset in Figure 2D), respectively. The carbon derived from the precursor itself is high in content, which can be improved by high-temperature carbonization. Additionally, the N content in the resultant NiCo/CN-800 is up to 5.32%, which could enhance the extent of defects, increase the active site and promote the catalytic activity (Xia et al., 2015; Liu and Dai, 2016; You et al., 2016). The presence of Ni and Co provides strong evidence for successful bimetallic doping. In general, the Co and Ni sites are regarded as the active species for ORR and OER, respectively. In particular, the interaction between Ni and Co can further improve the oxygen-evolving reactions.

**Figure 3:**
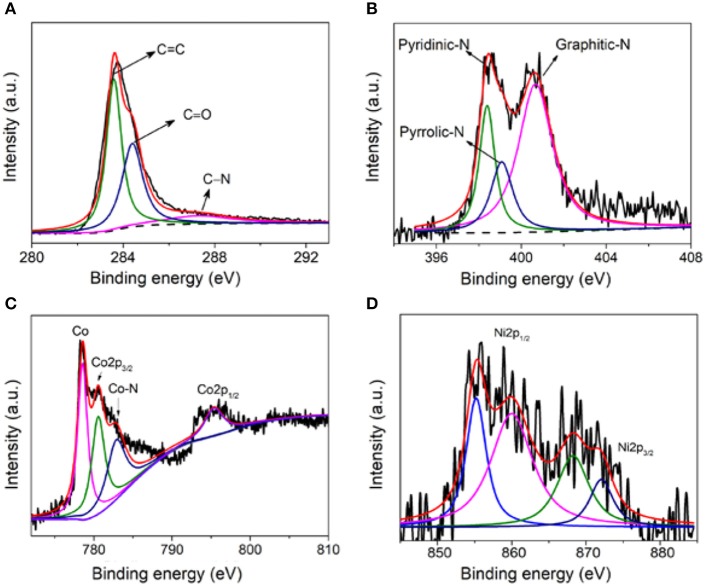
**(A)** XPS C1s binding energy spectrum of NiCo/CN-800, **(B)** N1s binding energy spectrum, **(C)** Co2p binding energy spectrum, and **(D)** Ni binding energy spectrum.

In addition, the specific chemical states of C and N in the sample were also analyzed. Since carbon is the main component of the prepared non-precious carbon-based materials, the high-resolution XPS spectrum of C1s was firstly analyzed in Figure 3A. The C1s XPS spectrum exhibits several characteristic peaks at 284.8, 286.9, and 288.5 eV, corresponding to C = C, C–N, and C = O, respectively. The N1s XPS spectrum in Figure 3B shows three characteristic peaks at 398.7, 400.15, and 401.3 eV, corresponding to N of pyridine, pyrrole, and graphite, respectively. Figure 3C is the binding energy spectrum of Co2p, Co, Co2p_1/2_, Co2p_3/2_, and Co-N. It is further confirmed that nitrogen also successfully binds to cobalt and increases more active sites (Dominguez et al., 2010; Hou et al., 2012; He et al., 2013; Su et al., 2014). On the other hand, in Figure 3D, the four peaks located at 855.2, 860.1, 868.2, and 872.2 eV show the exitance of metallic Ni, Ni2p_1/2_, Ni2p_3/2_, and Ni–N, demonstrating the coexistence of Ni and Co elements (Pu et al., 2016; Li Y. et al., 2019).

### Electrochemical Characterization of NiCo/CN-800

**Figure 4:**
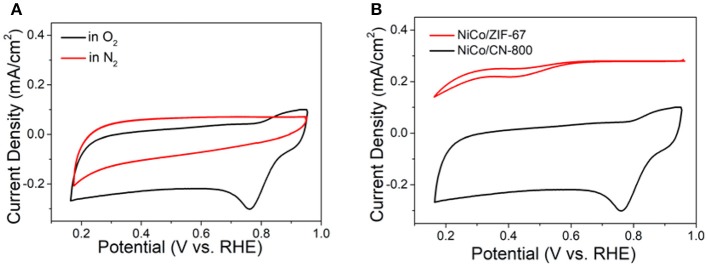
CV curves in the 0.1 M KOH. **(A)** CV curves of the carbonized material in O_2_ (black) and N_2_-saturated solutions (red), respectively. **(B)** CV curves of the NiCo/CN-800 and NiCo/ZIF-67.

Subsequently, the electrochemical tests using the conventional three-electrode system were conducted to investigate the relationship between material structure and electrochemical performance. Figure 4 is the cyclic voltammetry curve (CV) of NiCo/CN-800 in a 0.1 M KOH solution at a sweep rate of 50 mV/s. Figure 4A shows the CV curve of NiCo/CN-800 under N_2_-saturated or O_2_-saturated atmosphere. NiCo/CN-800 has no obvious peak under the N_2_-saturated solution, whereas an obvious reduction peak appears at 0.78 V (vs. RHE) under O_2_-saturated solution, indicating a good catalytic activity for ORR. Figure 4B shows the CV curves of NiCo/ZIF-67 and NiCo/CN-800 under O_2_-saturated solution. As shown in Figure 4B, a well-defined O_2_ reduction peak centered at ca. 0.76 V (vs. RHE) emerges when the electrolyte solution is saturated with O_2_. As a comparison, under the identical experimental condition, a weak O_2_ reduction peak is located at 0.45 V for initial NiCo/ZIF-67, verifying that the oxygen reduction potential is greatly improved after converting ZIF-67 into NiCo/CN-800.

**Figure 5:**
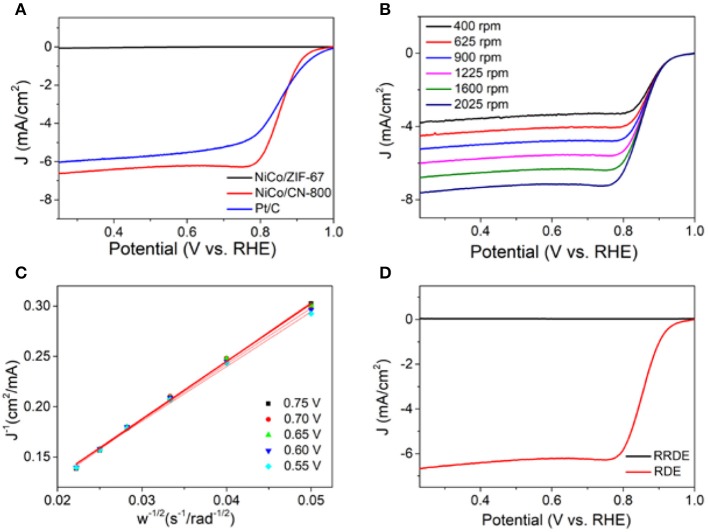
**(A)** LSV curves of NiCo/ZIF-67, NiCo/CN-800 and Pt/C (20 wt%) at a rotation rate of 1,600 rpm. **(B)** LSV curves of NiCo/CN-800 in O_2_-saturated solution with 0.1 M KOH of various rotation rate. **(C)** Koutecky–Levich plots of NiCo/CN-800 derived from LSV cures at different electrode potentials. **(D)** RRDE curves of the NiCo/CN-800.

To further explore the catalytic performance of NiCo/CN-800, we performed the electrocatalytic performance and electron transfer number of NiCo/CN-800 by using a rotating disk electrode and a rotating ring disk electrode. Figure 5A shows the LSV curve of NiCo/ZIF-67, NiCo/CN-800, and commercial Pt/C (20 wt%) tested in 0.1 M KOH aqueous solution with a rotation rate of 1,600 rpm. The diffusion-limited current densities of NiCo/ZIF-67, NiCo/CN-800, and commercial Pt/C (20 wt%) were 0.9, 6.7, and 6.1 mA/cm^2^, respectively. Compared with NiCo/ZIF-67, the ORR activity of NiCo/CN-800 significantly enhanced, indicating that the high-temperature carbonization is beneficial to enhancing the oxygen reduction performance. The initial potential of NiCo/CN-800 is 0.92 V, which is better than the reported NiCo-based ORR catalysts (Table S1) and even comparable to commercial Pt/C (0.96 V), and the half-wave potential of NiCo/CN-800 (0.86 V) is basically the same as that of commercial Pt/C (0.85 V). To further illustrate the ORR mechanism, the LSV curve with rotational speeds from 400 to 2,025 rpm on the RDE is shown in Figure 5B. For ORR, electron transfer number (*n*) is also an important parameter to measure the electrocatalytic performance. The corresponding Koutecky–Levich (K–L) plots (*J*^−1^ vs. ω^1/2^) were obtained according to the standard method (Figure 5C). The linear plots showed the first-order reaction kinetics toward oxygen (Mayrhofer et al., 2008). Based on the K-L equation, *n* of NiCo/CN-800 was 3.92 in the potential range from 0.55 to 0.75 V, which is very close to the ideal four-electron transfer process. Next, in order to further explain the electrocatalytic performance and better mass transfer studies, the kinetics of NiCo/CN-800 was also measured by a rotating ring-disk electrode (RRDE) under the same conditions in Figure 5D. The electron transfer number (*n*) of NiCo/CN-800 was calculated to be 3.95. Those result showed that the state of oxygen reduction on the NiCo/CN-800 electrode is approaching 100% of the four-electron transfer, and it also indicates that the material has excellent catalytic efficiency.

**Figure 6:**
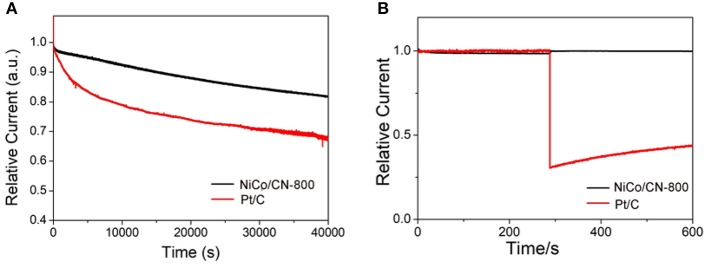
**(A)** Current-time (i-t) chronoamperometric responses at −0.6 V (vs. RHE) in O_2_-saturated 0.1 M KOH at NiCo/CN-800 and Pt/C electrodes (1,600 rpm) for 40,000 s. **(B)** Chronoamperometric responses at −0.6 V in O_2_-saturated 0.1 M KOH at NiCo/CN-800 or Pt/C electrodes (1,600 rpm) before and after addition of 1 M methanol.

In addition to catalytic activity, stability and methanol tolerance are also two key parameters for the practical application in fuel cells. The stability test of NiCo/CN-800 was shown in Figure 6A using a chronoamperometry at −0.6 V (vs. RHE) and in O_2_ saturated 0.1 M KOH electrolyte at 1,600 rpm. As a comparison, Pt/C (20 wt%) was also measured under the same experimental conditions. After 40,000 s, a current density loss at the NiCo/CN-800 catalyst was about 17%, and the corresponding loss at Pt/C was as high as 31%. This shows that the stability of the NiCo/CN-800 catalyst for ORR is better than that of the commercial Pt/C catalyst. Chronoamperometric responses of NiCo/CN-800 and Pt/C electrodes were investigated in Figure 6B. After injecting 1 M methanol at 300th s, the voltammetric current of the NiCo/CN-800 catalyst was nearly without change, whereas an observed current attenuation can be seen for commercial Pt/C, demonstrating the excellent selectivity of the as-prepared NiCo/CN-800 catalyst. These results indicate that the NiCo/CN-800 catalyst not only maintains high stability in an alkaline solution but also has high selectivity and outstanding methanol tolerance for ORR.

**Figure 7:**
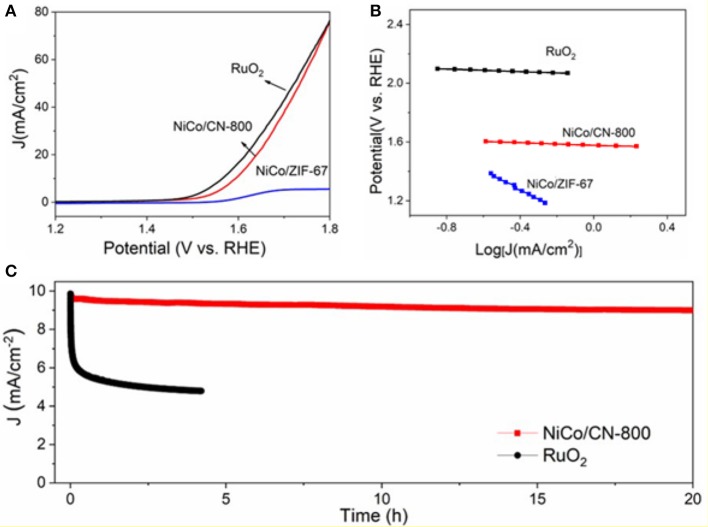
**(A)** LSV polarization curves of NiCo/CN-800 and NiCo/ZIF-67; **(B)** the corresponding Tafel plots. **(C)** Durability of the as-prepared NiCo/CN-800 and commercial RuO_2_ under the high current density of 10 mA/cm^−2^.

The electrode for OER performance evaluation was prepared by homogeneously depositing 0.25 mg cm^−2^ of as-synthesized NiCo/CN-800 onto a glassy carbon (GC) supporting electrode. The initial measurements were carried out in a standard three-electrode cell containing 1.0 M KOH solution at a scan rate of 10 mV s^−1^ to minimize capacitive background current. In Figure 7A, a sharply increased anodic current response started at an onset potential (*E*_onset_) of 1.48 V (defined as a potential required for reaching an OER current density of 0.2 mA cm^−2^) is observed from the NiCo/CN-800 electrode, indicating a markedly improved catalytic activity compared to that of the NiCo/ZIF-67. Besides the *E*_onset_, the operational potential at 10 mA cm^−2^ (*E*_j = 10_) is another key parameter for OER performance evaluation. When the thermodynamic OER potential $\left({{\text{E}}^{0}}_{\text{H}2\text{O/O}2}=1.229\text{ V}\right)$ is used as the reference, the NiCo/CN-800 electrode possesses a low overpotential of ~350 mV at 10 mA cm^−2^, which is very close to that of the noble metal (RuO_2_) catalysts (~330 mV). Interestingly enough, the overpotential of NiCo/CN-800 at a high current density of 80 mA cm^−2^ is the same as that of the commercial RuO_2_, which is ascribed to the excellent gas diffusion and mass transfer of the porous NiCo/CN-800. Subsequently, the catalytic kinetics of NiCo/CN-800 is assessed by the Tafel plots in an O_2_-saturated 1 M KOH solution. As displayed in Figure 7B, the resultant Tafel slope of NiCo/CN-800 (65 mV dec^−1^) is much smaller than that of NiCo/ZIF-67 (155 mV dec^−1^) and comparable to the commercial RuO_2_ (60 mV dec^−1^), signifying a superior reaction kinetics (Lee et al., 2012; Aijaz et al., 2016). Other than the catalytic activity, the robustness and durability are also key parameters to evaluate the performance of the catalyst. As shown in Figure 7C, under a constant current density of 10 mA/cm^−2^, only 8.6 % decay in anodic current is observed after 20 h of continuous electrolysis, which is obviously superior to that of the commercial RuO_2_ catalyst. The excellent stability should be mainly attributed to the porous structure of the as-prepared NiCo/CN-800.

## Conclusions

In summary, a series of N-doped bimetallic metal-carbon hybrid catalysts (NiCo/CNs) derived from NiCo/ZIF-67 were successfully prepared by one-step pyrolysis for use as an efficient bifunctional electrocatalyst for both ORR and OER. The structural merits of the NiCo/CN-800 catalysts were revealed by N_2_ adsorption isotherm, XRD, XPS, and Raman spectroscopy. The resultant N-doped mesoporous NiCo/CN-800 exhibits superior catalytic activity and excellent durability as a bifunctional ORR and OER catalyst, which is close to and, in some cases, even better than that of noble metal catalysts (Pt/C and RuO_2_/C). The active dopants, porous structure and the good electron transfer ability are believed to significantly expedite the ORR and OER processes. This proof-of-concept study lays a solid foundation for the further exploration and development of nanostructured MOF derivatives for electrocatalysis and energy conversion applications such as metal-air batteries and supercapacitors.

## Data Availability Statement

All datasets generated for this study are included in the article/Supplementary Material.

## Author Contributions

ZW, CT, and K-HW proposed the research direction and guided the project. YZho and YZha designed and performed the experiments. YZho, YZha, and XX analyzed and discussed the experimental results and drafted the manuscript. YZho, YZha, ZG, and SZ joined the discussion of data and gave useful suggestions.

### Conflict of Interest

The authors declare that the research was conducted in the absence of any commercial or financial relationships that could be construed as a potential conflict of interest.
