# Supplementary material for: Bimetallic Metal-Organic Framework Derived Metal-Carbon Hybrid for Efficient Reversible Oxygen Electrocatalysis
Source: Front Chem. 2019 Nov 8;7:747. doi: 10.3389/fchem.2019.00747 (PMC6856206; doi:10.3389/fchem.2019.00747)
Supplement: Supplementary file 1 [file Data_Sheet_1.PDF]

Supporting Information for “imetallic Metal-Organic Framework Derived Metal-Carbon Hybrid for Efficient Reversible Oxygen Electrocatalysis”

Yu Zhou,[a] Yan Zhang,[a] Xianzhen Xu,[a] Shenlong Zhao[c] Ziyi Guo[b], Kuang-Hsu Wu*[b], Chunhui Tan*[b], Zonghua Wang,*[a]

a Shandong Sino-Japanese Center for Collaborative Research of Carbon Nanomaterials, College of Chemistry and Chemical Engineering, Qingdao University, Qingdao 266071, China.

b School of Chemical Engineering, the University of New South Wales, Sydney, Kenginton NSW 2052, Australia.

c School of Chemical and Biomolecular Engineering, The University of Sydney, Sydney, New South Wales, 2006, Australia

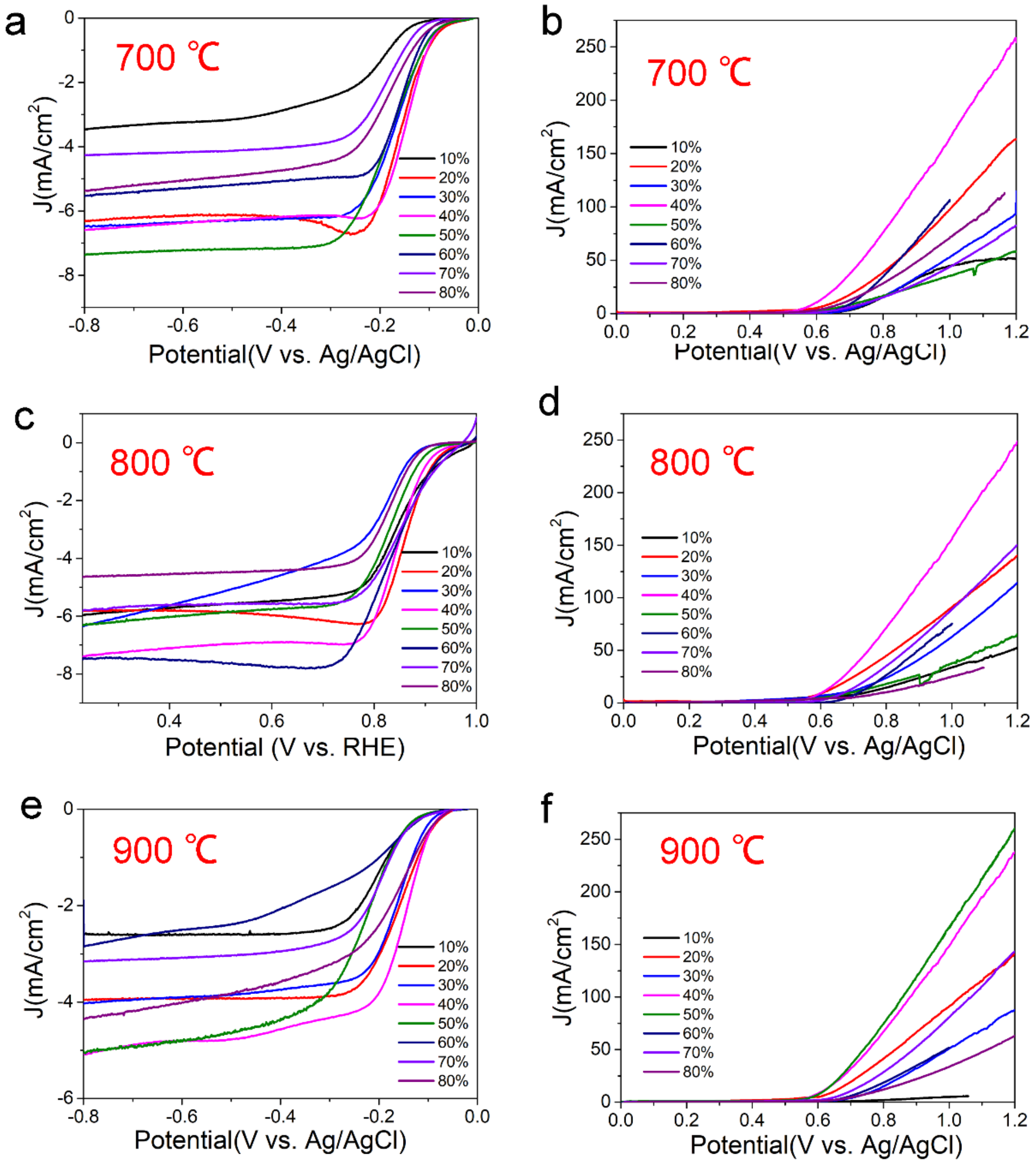

Figure S1. LSV curves of NiCo/CN at different doping ratio and different pyrolysis temperature. The corresponding doping ratio and pyrolysis temperature were shown in the figure.

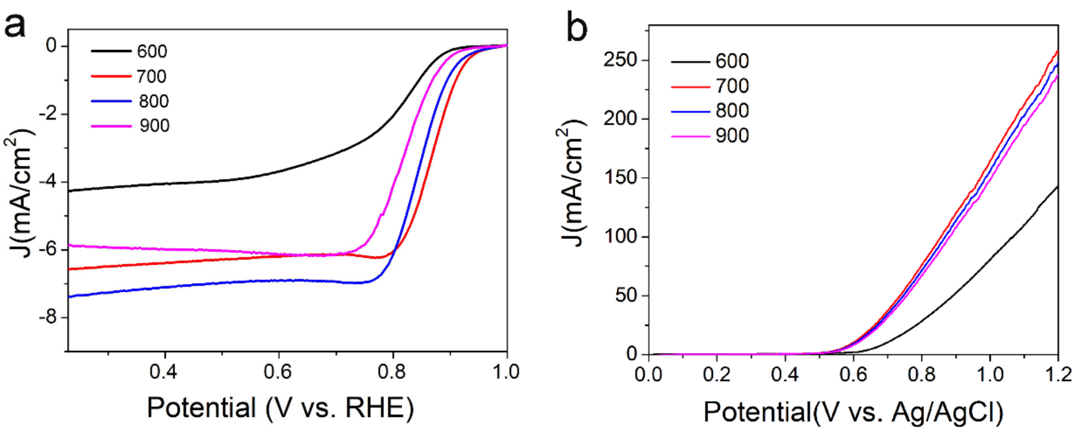

Figure S2. LSV curves of NiCo/CN at different temperatures.

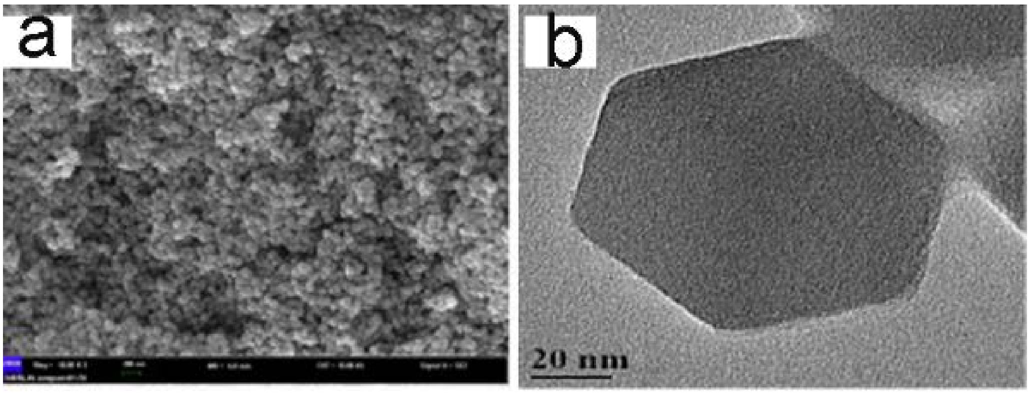

Figure S3. SEM (a) and TEM (b) image of ZIF-67

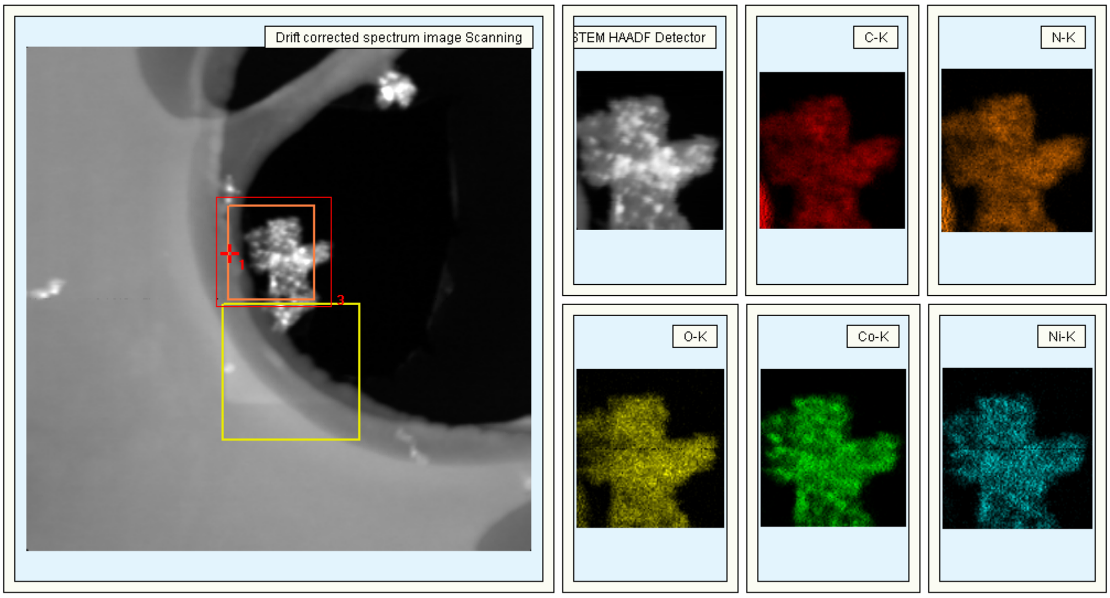

Figure S4. EDX maping of C, N, O, Co, Ni in NiCo/CN-800

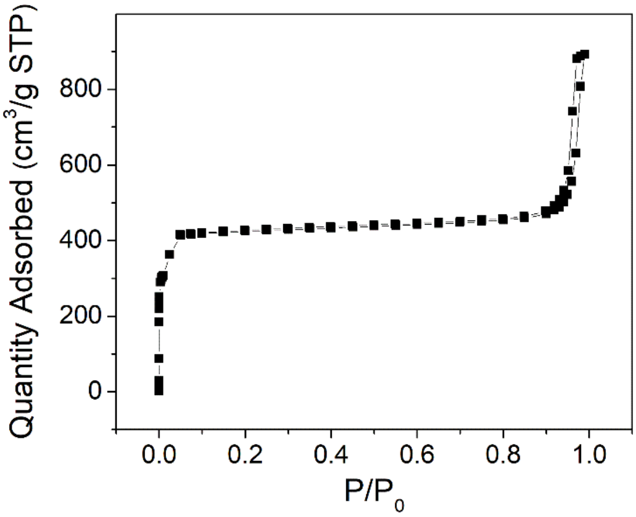

Figure S5. N_2 adsorption-desorption isotherm of the carbonized product of NiCo/ZIF-67.

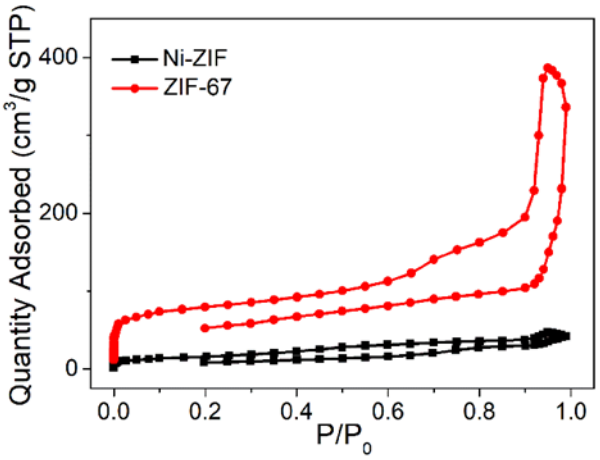

Figure S6. N_2 adsorption-desorption isotherm of the carbonized product of (a) Ni-ZIF and (b) ZIF-67. The specific surface area of Ni-ZIF and ZIF-67 was found to be 70, 392 m^2/g, respectively.

Material	Current densities in ORR (mA cm^{-2})	ORR onset (V)	OER onset (V)	Reference
NiCo@N-C 2	6.54	0.96	1.76	Fu et al., 2017
N-G@CoNi/BCNT	~6.7		-	Hou et al., 2016
NPCN/CoNiNCNT	~6.0	0.94	1.59	Hou et al., 2015
Ni–Co hydroxide	-	-	1.69	Zhao et al., 2014
Ni-doped Co_3O_4 NW	5.8	~0.94	-	Tong et al, 2015
Co–Ni LDHs	-	-	1.623	Zhang et al., 2013
CoNi(OH)x	-	-	1.48	Li et al., 2016
NiCo/CN-800	6.7	0.96	1.48	This work

Table S1. Performance comparison of the NiCo/CN-800 with the reported NiCo-based electrocatalysts for OER and ORR.

Fu, Y., Yu, H. Y., Jiang, C., Zhang, T. H., Zhan, R., Li X. W., et al. (2017). NiCo Alloy Nanoparticles Decorated on N-Doped Carbon Nanofibers as Highly Active and Durable Oxygen Electrocatalyst. *Adv. Funct. Mater.* 28. 1705094.

Hou, Y., Yuan, H., Wen, Z. H., Cui, S. M., Guo, X. R., He, Z., et al. (2016). Nitrogen-Doped Graphene/CoNi Alloy Encased Within Bamboo-Like Carbon Nanotube Hybrids as Cathode Catalysts in Microbial Fuel Cells. *J. Power Sources.* 307. 561-568.

Hou, Y., Cui, S., Wen, Z., Guo, X., Feng, X., and Chen J. (2015) Strongly Coupled 3D Hybrids of N-doped Porous Carbon Nanosheet/CoNi Alloy-Encapsulated Carbon Nanotubes for Enhanced Electrocatalysis. *small*. 11. 5940–5948.

Zhao, Z., Wu, H., He, H., Xu, X., Jin, Y. (2014) A High-Performance Binary Ni–Co Hydroxide-based Water Oxidation Electrode with Three-Dimensional Coaxial Nanotube Array Structure. *Adv. Funct. Mater.* 24. 4698-4705.

Tong, X., Xia, X., Guo, C., Zhang, Y., Tu, J., Fan, H., et al. (2015) Efficient oxygen reduction reaction using mesoporous Ni-doped Co3O4 nanowire array Electrocatalysts. J. Mater. Chem. A. 3. 18372-18379.

Zhang, Y., Cui, B., Zhao, C., Lin, H. and Li, J. (2013) Co–Ni layered double hydroxides for water oxidation in neutral electrolyte. Phys.Chem. Chem. Phys. 15. 7363-7369.

Li, S., Wang, Y., Peng, S., Zhang, L., Al-Enizi, A. M., Zhang, H., et al (2016) Co-Ni based Nanotubes/Nanosheets as Efficient Water Splitting Electrocatalysts. Adv. Energy Mater. 6. 1501661
